# Cluster synchronization for controlled nodes via the dynamics of edges in complex dynamical networks

**DOI:** 10.1371/journal.pone.0288657

**Published:** 2023-08-03

**Authors:** Lizhi Liu, Cao Chen, Zilin Gao, Bo Cheng

**Affiliations:** 1 School of Information Science and Engineering, Hunan Institute of Science and Technology, Yueyang, Hunan, China; 2 School of Computer Science and Engineering, Chongqing Three Gorges University, Chongqing, China; 3 College of Automation, Chongqing University of Posts and Telecommunications, Chongqing, China; University of Shanghai for Science and Technology, CHINA

## Abstract

An appropriate dynamic coupling form between nodes and edges’ state can effectively promote the emergence of desired network function (phenomenon), but the existing literatures have not conducted in-depth research on the coupling mechanism. This paper mainly focuses on the coupling auxiliary mechanism of dynamic edges for the emergence of cluster phenomenon of nodes, explores the essential relation between structure and function in complex dynamical networks (CDNs). Firstly, a novel model of CDNs has dynamic systems attached on not only nodes but also edges is proposed from the viewpoint of large-scale system. Secondly, a feedback nodes controller is synthesized associate with the designed linear and adaptive dynamics of edges. Via the appropriate dynamic behaviors of the edges system, the controlled nodes can realize cluster synchronized. Finally, the validity of the proposed approaches is verified by a given numerical example.

## Introduction

In early two decades, cluster synchronization in complex dynamical networks (CDNs) has been widely studied because of its application in communication engineering and bioscience [1–4]. From the viewpoint of graph theory, the nodes and edges, as the constituent elements of complex network, represent respectively the entities and connection relationships between entities in real networks. The nodes are seen as the representation subject of cluster synchronization, the edges are regarded as the bridge of communication between nodes to assist the nodes realizing the desired cluster phenomenon via specific connection mode and strength [5].

In order to more truly depict the structure of real networks, some types of edges are utilized to describe the topology of CDNs in the existing literatures, such as, fixed topologies [6, 7], multiple links [8–10], switchable topologies [11–13], random connection [14, 15] and so on. Multi-links mean that there exists more than one edge between nodes, for example, people can connect each other by mail, telephone, e-mail and so on. Switchable topologies imply that there are multiple different topologies in complex network controlled by switching signals. In the studies about CNDs with random edges, the change of connection (connect or not-connect) and strength obeys the given probability distribution. Although the diversity of edges and its impact on the realization of cluster synchronization have been considered as noted above literatures, the edges are not seen as the dynamic systems just like the nodes.

In many researches on dynamic characteristics in real networks, the edges are regarded as the dynamic systems like the node, the mutual-coupling dynamic behaviors between nodes and edges are studied. For example, in Internet congestion control, the resource transmission rate of competing users and the links’ price can be regarded as the state of nodes and edges, respectively. By designing the appropriate dynamics of edges, the maximum utility can be obtained [16, 17]. In industrial web-winding system, the coupling mathematical models about motors’ (nodes) velocity and webs’ (edges) tension are formed, the both can track the reference value via the proposed controller [18, 19]. In biological nerve researches, some dynamics of edges are proposed to understand learning, memory and oscillations in neural network [20–25]. The edges can be provided with dynamic coupling characteristics with nodes instead of static or simple switchable from the above instances, the desired function of complex network will appear when the edges present the appropriate dynamic behavior. However, the dynamics of dynamic edges for the evolution of cluster synchronization is discussed rarely.

In recent researches about CDNs control, the mutual-coupling models with nodes and edges are proposed to explore the dynamic behaviors of CDNs. In [26, 27], the matrix equations about the connection relationships between nodes are built to analyze the evolution process of structural balance, the nodes can be divided into two antagonistic factions in the complex networks satisfying the feature of structural balance. In [28, 29], the dynamic behaviors of edges are concerned further, the Riccati-type matrix differential or difference equations are adopted as the mathematical model of edges, the tracking control goal of edges can be realized via the help of controlled nodes. In [30–32], the complete synchronization of nodes is achieved via the assistant role of the proposed dynamics of edges.

Through the further analysis of the existing literatures, the shortcomings are summarized as follows: (i) In [6–9, 11, 12, 14, 15], only one of the nodes and edges’ model is established not both of them. (ii) In [28–32], the cluster synchronization of the CDNs composed of the dynamic edges and nodes is not discussed, just pay attention on complete synchronization. (iii) In [30, 32], the presupposed synchronization state of nodes is strongly related to the tracking target of edges, which is not common. In order to compensate for these deficiencies, firstly, this paper proposes the nodes and edges dynamic models which are coupled mutually. Then, by designing the more universal coupling form and node controllers, we explore the cluster synchronization of nodes under the auxiliary role of the designed edges’ dynamics of edges, and discuss the different roles that the linear and adaptive dynamics of edges for the dynamic behaviors of CDN.

**Fig 1:**
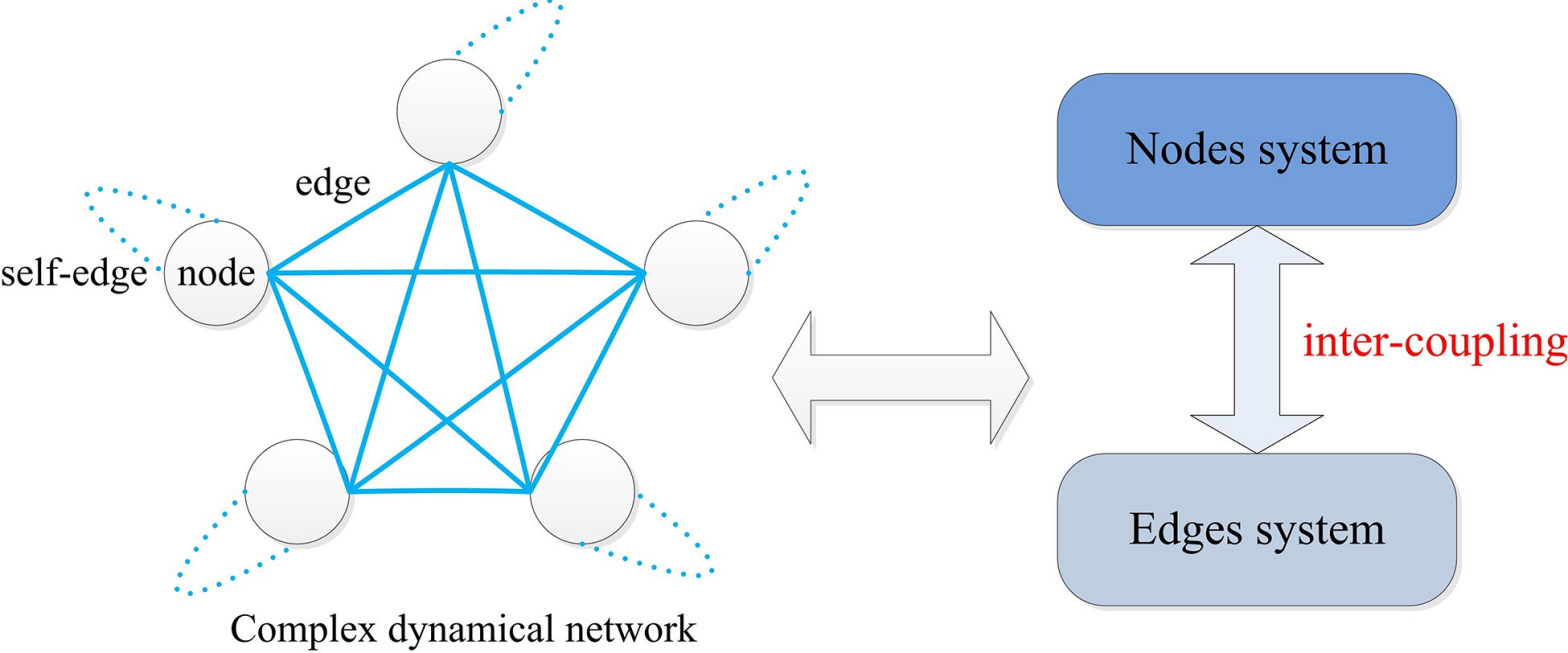
The model composed of the edges and nodes systems.

The main contributions are as follows: (i) Compared with [6–9, 11, 12, 14, 15], the novel model of continuous-time CDNs is proposed. From the viewpoint of large-scale system control theory, the CDNs are considered to be composed of the nodes and edges dynamical systems (see Fig 1), the mutual-coupling mathematical models are established at the same time not just nodes or edges. (ii) Two equations are employed to govern the dynamic behaviors of linear and adaptive edges systems. The cluster synchronization is explored, and the different effects of linear and adaptive dynamics of edges for the dynamic behaviors of CDNs are discussed. Compared with [28–32], the simplicity and universality of designed coupling form are more than the counterpart. (iii) Under the proposed adaptive dynamics of edges, the interconnection in the same cluster does not tend to zero while the synchronization is achieved, this result is more general compared to the existing literatures.

The remainder of the paper is organized as follows. Section 2 gives the mathematical model and preliminaries. Section 3 presents the dynamics of edges system and the design approach of node controller. A numerical simulation and conclusion are given in section 4, 5.

### Preliminaries and model description

**Notations:** *A*^*T*^, *A*^−1^ denote the transpose and inverse of the matrix *A*, respectively. ‖⋅‖ denotes the Euclid norm of the vector or the matrix. *R*^*n*^, *R*^*n*×*n*^ denote the *n*-dimensional Euclidean space and the set of *n*×*n* real matrix, respectively. *I*_*n*_ denotes the *n*-dimensional identity matrix. *diag*{⋯} stands for a diagonal matrix or blocked diagonal matrix. *A*<0(>0) denotes matrix *A* is a negative (positive) definite matrix (all real parts of eigenvalues are negative (positive)). Let *A* = [*a*_*ij*_]_*n*×*n*_, defining the operator *D*[*A*] = [*d*_*ij*_]_*n*×*n*_, if *a*_*ij*_≠1 then *d*_*ij*_ =1, otherwise *d*_*ij*_ = 0. Symbols ⊗, ∘ stand for the Kronecker product and Hadamard product, defined as $A⊗B=[\begin{array}{ccc} a_{11}B & \cdots & a_{1m}B\\ \vdots & \ddots & \vdots \\ a_{n1}B & \cdots & a_{nm}B \end{array}]$, $A\circ C=[\begin{array}{cccc} a_{11}c_{11} & a_{12}c_{12} & \cdots & a_{1m}c_{1m}\\ a_{21}c_{21} & a_{22}c_{22} & \cdots & a_{2m}c_{2m}\\ \vdots & \vdots & \ddots & \vdots \\ a_{n1}c_{n1} & a_{n2}c_{n2} & \cdots & a_{nm}c_{nm} \end{array}]$, where *B* is a matrix of any dimension, *A* = [*a*_*ij*_]_*n*×*m*_, *C* = [*c*_*ij*_]_*n*×*m*_.

Consider the continuous-time CDNs with *N* nonidentical nodes, the dynamic equations of controlled nodes are proposed as follows

$${\dot{x}}_{i}(t)=f_{i}(x_{i}(t))+c\sum_{j=1}^{N}a_{ij}(t)\Gamma h_{j}(x_{j}(t))+u_{i}(t),i=1,2,\cdots ,N,$$ (1)

where, *x*_*i*_(*t*)∈*R*^*n*^ is the state vector of nodes *i*, *f*_*i*_(⋅):*R*^*n*^→*R*^*n*^ is nonlinear bounded function, *c*∈*R* is common coupling strength, Γ∈*R*^*n*×*n*^ is inner coupled matrix, *h*_*j*_(⋅):*R*^*n*^→*R*^*n*^ is coupled function and *u*_*i*_(*t*) is control input of node *i*. *a*_*ij*_ = *a*_*ij*_(*t*)∈*R* denote the time-varying connection weight (edges) from node *j* pointing to node *i*.

Let $x(t)={\left[x_{1}(t),x_{2}(t),\cdots ,x_{N}(t)\right]}^{T}$, $A=A(t)={\left[a_{ij}(t)\right]}_{N\times N}$, the nodes system composed of all nodes is described by the following equation via Kronecker product

$$\dot{x}(t)=F(x(t))+c[A(t)⊗\Gamma ]H(t)+u(t),$$ (2)

where $F(x(t))={\left[f_{1}^{T}(x_{1}(t)),f_{2}^{T}(x_{2}(t)),\cdots ,f_{N}^{T}(x_{N}(t))\right]}^{T}$, $H(t)={\left[h_{1}^{T}(x_{1}(t)),h_{2}^{T}(x_{2}(t)),\cdots ,h_{N}^{T}(x_{N}(t))\right]}^{T}$, and $u(t)={\left[u_{1}{\left(t\right)}^{T},u_{2}{\left(t\right)}^{T}\cdots ,u_{N}{\left(t\right)}^{T}\right]}^{T}$.

Define the edges system as follows

$${\dot{a}}_{ij}(t)=\{\begin{array}{c} \phi_{ij}(A(t),x(t)),\mathrm{node}\;i\;\mathrm{connects}\;\mathrm{directly}\;\mathrm{node}\;j,\\ 0,\mathrm{node}\;i\;\mathrm{connects}\;\mathrm{undirectly}\;\mathrm{node}\;j, \end{array}$$ (3)

where, function $\phi_{ij}(\cdot )\neq \phi_{ji}(\cdot )$ (i,j=1,2,⋯,N) which implies the network is directed, especially, the self-edge *a*_*ii*_(*t*) is considered.

Let vectors $a_{i}(t)={\left[a_{i1}(t),a_{i2}(t),\cdots ,a_{iN}(t)\right]}^{T}(i=1,2,\cdots ,N)$ and $\tilde{A}(t)=[a_{1}{}^{T}(t),$ $a_{2}{}^{T}(t),\cdots ,a_{N}{}^{T}(t){]}^{T}$, the mathematical model of the node system can be rewritten as follows

$$\dot{x}(t)=F(x(t))+c[I_{N}⊗(\Gamma \tilde{H}(t))]\tilde{A}(t)+u(t),$$ (4)

where $\tilde{H}(t)=[h_{1}(x_{1}(t)),h_{2}(x_{2}(t)),\cdots ,h_{N}(x_{N}(t))]$.

**Remark 1.** (i) By the Vectorization operator $vec(A)={\left[a_{11},\cdots ,a_{n1},a_{12},\cdots ,a_{n2},\cdots ,a_{1m},\cdots ,a_{nm}\right]}^{T}$, the equation $vec(BAC)=(C^{T}⊗B)vec(A)$ holds, where *A* = [*a*_*ij*_]_*n*×*m*_, *B*∈*R*^*s*×*n*^, *C*∈*R*^*m*×*g*^. Further, we can obtain $\left[A(t)⊗\Gamma ]H(t)=vec(\Gamma \tilde{H}(t)A{\left(t\right)}^{T})=[I_{N}⊗(\Gamma \tilde{H}(t))]vec(A{\left(t\right)}^{T})=[I_{N}⊗(\Gamma \tilde{H}(t))]\tilde{A}(t\right)$, thus, we can derive Eq (4) from Eq (2). (ii) The proposed complex networks in this paper have dynamic systems attached on not only nodes but also edges, therefore, the dynamic behavior of edges cannot be ignored in the evolution analysis of cluster synchronization of nodes. (iii) In general, the exact values of the edges’ state are not obtained without state observer or sensor, which implies the state of edges is unavailable for controller *u*(*t*) and is not controlled directly.

**Assumption 1.** If function *f*_*i*_(*x*_*i*_(*t*)) (*i* = 1,2,⋯,*N*) is norm bounded, there are exist *N* known bounded nonnegative continuous functions *θ*_*i*_(*x*_*i*_(*t*),*t*) (*i* = 1,2,⋯,*N*) satisfy that $\left\|f_{i}(x_{i}(t))\|\leq \theta_{i}(x_{i}(t),t\right)$.

**Assumption 2.** The coupled functions *h*_*j*_(*x*_*j*_(*t*)) (*j* = 1,2,⋯,*N*) are bounded.

**Lemma 1 [33].** The following properties are true via Kronecker product:

1. (A⊗B)+(A⊗C)=A⊗(B+C),
2. (A⊗B)(C⊗D)=(AC)⊗(BD),
3. ${\left(A⊗B\right)}^{T}=A^{T}⊗B^{T}$,

where *A*,*B*,*C* and *D* are matrices with appropriate dimensional.

**Definition 1.** Let {*G*_1_,*G*_2_,⋯,*G*_*M*_} be a partition of the set {1,2,⋯,*N*} into *M*nonempty subsets, that is, $\overset{M}{\underset{k=1}{\cup }}G_{k}=\{1,2,\cdots ,N\}$ and *G*_*k*_≠∅. If the $\underset{t\to \infty }{\lim}\|x_{i}(t)-x_{j}(t)\|=0$ for *i*,*j*∈*G*_*k*_, the complex network with systems (3) and (4) is said to achieve the cluster synchronization.

**Control goal.** Consider the CDNs with the edges system (3) and nodes system (4), design the appropriate nodes controller *u*(*t*) and dynamics of edges *ϕ*_*ij*_(⋅) such that the cluster synchronization can be realized.

**Fig 2:**
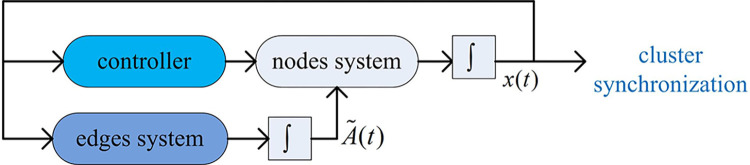
The control diagram of the two intercoupling systems.

In order to clarify the control architecture of this paper, the following diagram is given in Fig 2.

### Main results

In this section, we propose two dynamics of edges to explore the cluster synchronization of CDNs, respectively.

We suppose *N* nodes achieve *M*-cluster synchronization and the whole nodes splits into *M* clusters. Without loss of generality, the sets of subscripts of these clusters are $G_{1}=\{1,2,\cdots ,N_{1}\}$, $G_{2}=\{N_{1}+1,N_{2}+2,\cdots ,N_{1}+N_{2}\},\cdots ,G_{M}=\{N_{1}+\cdots +N_{M-1}+1,\cdots ,N\}$. Define synchronous errors in cluster *G*_*j*_ (*j* = 1,2,⋯,*M*), $e_{j,k_{j}}(t)=x_{s_{j}}(t)-x_{s_{j}+1}(t)$ ($k_{j}=1,2,\cdots ,N_{j}-1$), $e_{j,N_{j}}(t)=x_{l_{j}}(t)-x_{1+l_{j-1}}(t)$, where $s_{j}=(1+l_{j-1})+(2+l_{j-1})+\cdots +(l_{j}-1)$, $l_{j}=\sum_{j=1}^{j}N_{j}$ (*N*_0_ = 0). Next, we introduce the following constant matrix Λ_*j*_ (*j* = 1,2,⋯,*M*)

$$\Lambda_{j}=(\begin{array}{cccccc} 1 & -1 & 0 & \cdots & 0 & 0\\ 0 & 1 & -1 & \cdots & 0 & 0\\ 0 & 0 & 1 & \cdots & 0 & 0\\ \vdots & \vdots & \vdots & \ddots & \vdots & \vdots \\ 0 & 0 & 0 & \cdots & 1 & -1\\ -1 & 0 & 0 & \cdots & 0 & 1 \end{array})\in R^{N_{j}\times N_{j}}.$$ (5)

**Remark 2.** (i) Let $X(t)={\left[x_{1}(t),x_{2}(t),\cdots ,x_{N}(t)\right]}^{T}$, $E_{j}(t)=[e{\left(t\right)}_{j,1},\cdots ,e{\left(t\right)}_{j,N_{j}}]$ (1≤*j*≤*M*), $E(t)={\left[E_{1}(t),E_{2}(t),\cdots ,E_{M}(t)\right]}^{T}$, $\Lambda =diag\{\Lambda_{1},\;\Lambda_{2},\cdots ,\Lambda_{M}\}$, it can derive that *E*(*t*) = Λ*X*(*t*). Matrix Λ is singular which implies $\underset{t\to \infty }{\lim}X(t)=0$ cannot be obtained directly from $\underset{t\to \infty }{\lim}E(t)=0$, thus, the cluster synchronization is non-trivial. (ii) Especially, if *M* = 1 the cluster synchronization will be complete synchronization.

**Theorem 1.** Consider the fully connected complex dynamical network with dynamics (3) and (4). If the Assumptions 1, 2 are held, the cluster synchronization of nodes system can be achieved via the proposed controller (6) and the dynamics of edges (7).

$$u(t)=[(\beta I_{N}+\gamma \Lambda^{+}\Lambda )⊗I_{n}]x(t)-\|\theta (x(t))\|sg(\Lambda_{\alpha }{}^{T}\Lambda_{\alpha }x(t)),$$ (6)

$${\dot{a}}_{i}(t)=P_{i}a_{i}(t)-cD[a_{i}(0)]\circ H^{T}(x(t))X^{T}(t)\Lambda^{T}{\left(\Lambda^{T}\right)}_{i}{}^{T},i=1,2,\cdots ,N,$$ (7)

where, *β* and *γ* are adjustable scalar that satisfy *β*+*γ*<0. *P*_*i*_∈*R*^*N*×*N*^ satisfies $P_{i}+P_{i}^{T}<0$, Λ^+^ is the Moore-Penrose inverse matrix of Λ, Λ_*α*_ = Λ⊗*I*_*n*_, (Λ^*T*^)_*i*_ is the *i*-th row of Λ^*T*^, $sg(x(t))=\{\begin{array}{c} \frac{x(t)}{\left\|x(t)\right\|},x(t)\neq 0\\ 0,x(t)=0 \end{array}$, $D[a_{i}(0)]={\left[d_{ij}\right]}_{n\times 1}$, if *a*_*ij*_(0)≠1 then *d*_*ij*_ = 1, otherwise *d*_*ij*_ = 0. If node *j* is not directly connected to node *i*, *a*_*ij*_(0) = 0, the *j*-th row of *P*_*i*_ is zero.

**Proof.** Define the cluster synchronization error vector $e(t)={\left[e_{1,1}^{T}(t),\cdots ,e_{1,N_{1}}^{T}(t),e_{2,1}^{T}(t),\cdots ,e_{2,N_{2}}^{T}(t),\cdots ,e_{M,1}^{T}(t),e_{M,N_{M}}^{T}(t)\right]}^{T}$. By the Vectorization operator, it can derive $e(t)=vec(E^{T}(t))=vec(X^{T}(t)\Lambda^{T})=\Lambda_{\alpha }x(t)$. Via the controller (6), the dynamical equation of cluster synchronization error *e*(*t*) can be expressed as follows

$$\begin{array}{l} \dot{e}(t)=\Lambda_{\alpha }\dot{x}(t)=\Lambda_{\alpha }\{F(x(t))+c[I_{N}⊗(\Gamma \tilde{H}(t))]\tilde{A}(t)+u(t)\}\\ \;=\Lambda_{\alpha }F(x(t))+c(\Lambda ⊗I_{n})[I_{N}⊗(\Gamma \tilde{H}(t))]\tilde{A}(t)\\ \;+\gamma (\Lambda ⊗I_{n})((\Lambda^{+}\Lambda )⊗I_{n})x(t)\\ \;+\beta e(t)-\|\theta (x(t))\|(\Lambda ⊗I_{n})sg(\Lambda_{\alpha }{}^{T}\Lambda_{\alpha }x(t))\\ \;=\Lambda_{\alpha }F(x(t))+c[\Lambda ⊗(\Gamma \tilde{H}(t))]\tilde{A}(t)+(\beta +\gamma )e(t)\\ \;-\|\theta (x(t))\|(\Lambda ⊗I_{n})sg(\Lambda_{\alpha }{}^{T}\Lambda_{\alpha }x(t)) \end{array}$$ (8)

Let matrices $\Lambda_{\beta }={\left[{\left(\Lambda^{T}\right)}_{1},{\left(\Lambda^{T}\right)}_{2},\cdots ,{\left(\Lambda^{T}\right)}_{N}\right]}^{T}$, $P=diag\{P_{1},P_{2},\cdots ,P_{N}\}$, the Equation is rewritten as follows:

$$\dot{\tilde{A}}(t)=P\tilde{A}(t)-c{\left[D[\tilde{A}(0)]\right]}^{T}[I_{N}⊗({\tilde{H}}^{T}(t)\Gamma^{T}X^{T}(t)\Lambda^{T})]\Lambda_{\beta }$$ (9)

Consider the following Lyapunov candidate function

$$V_{1}(t)=\frac{1}{2}[e^{T}(t)e(t)+\tilde{A}{\left(t\right)}^{T}\tilde{A}(t)].$$ (10)

The derivative of *V*_1_(*t*) along (8), (9) can be derived as

$$\begin{array}{l} {\dot{V}}_{1}(t)=e^{T}(t)\dot{e}(t)+\tilde{A}(t{)}^{T}\dot{\tilde{A}}(t)=(\beta +\gamma )e^{T}(t)e(t)+e^{T}(t)[\Lambda_{\alpha }F(x(t))\\ \;-\|\theta (x(t))\|\Lambda_{\alpha }sg(\Lambda_{\alpha }{}^{T}\Lambda_{\alpha }x(t))]+ce^{T}(t)[\Lambda ⊗\Gamma \tilde{H}(t)]\tilde{A}(t)+\tilde{A}(t)TP\tilde{A}(t)\\ \;-c{\tilde{A}}^{T}(t)\{[D[\tilde{A}(0)]{]}^{T}\circ [I_{N}⊗{\tilde{H}}^{T}(t)\Gamma^{T}X^{T}(t)\Lambda^{T}]\Lambda_{\beta }\}, \end{array}$$ (11)

By the controller (6) and Assumption 1, one has that

$$\begin{array}{l} e^{T}(t)[\Lambda_{\alpha }F(x(t))-\|\theta (x(t))\|\Lambda_{\alpha }sg(\Lambda_{\alpha }{}^{T}\Lambda_{\alpha }x(t))]\\ =-\|\theta (x(t))\|e^{T}(t)\Lambda_{\alpha }sg(\Lambda_{\alpha }{}^{T}e(t))+e^{T}(t)\Lambda_{\alpha }F(x(t))\\ \leq -\|\theta (x(t))\|\frac{e^{T}(t)\Lambda_{\alpha }\Lambda_{\alpha }{}^{T}e(t)}{\left\|\Lambda_{\alpha }{}^{T}e(t)\right\|}+\|e^{T}(t)\Lambda_{\alpha }\|\|F(x(t))\|\\ \leq \|e^{T}(t)\Lambda_{\alpha }\|(\|F(x(t))\|-\|\theta (x(t))\|)\\ \leq 0. \end{array}$$ (12)

From the Lemma 1, we can obtain that

$$\begin{array}{l} {}[I_{N}⊗({\tilde{H}}^{T}(t)\Gamma^{T}X^{T}(t)\Lambda^{T})]\Lambda_{\beta }\\ =[I_{N}⊗({\tilde{H}}^{T}(t)\Gamma^{T}E^{T}(t))]\Lambda_{\beta }\\ =vec({\tilde{H}}^{T}(t)\Gamma^{T}E^{T}(t)[(\Lambda^{T}{)}_{1},(\Lambda^{T}{)}_{2},\cdots ,(\Lambda^{T}{)}_{N}]T)\\ =[[(\Lambda^{T}{)}_{1},(\Lambda^{T}{)}_{2},\cdots ,(\Lambda^{T}{)}_{N}]⊗({\tilde{H}}^{T}(t)\Gamma^{T})]vec(E^{T}(t))\\ =[\Lambda^{T}⊗({\tilde{H}}^{T}(t)\Gamma^{T})]e(t). \end{array}$$ (13)

Because the network is fully connected, $D[\tilde{A}(0)]=\underset{N^{2}}{\underset{︸}{{\left[\begin{array}{ccc} 1 & \ldots & 1 \end{array}\right]}^{T}}}$, it can derive that

$${\left[D[\tilde{A}(0)]\right]}^{T}\circ [I_{N}⊗({\tilde{H}}^{T}(t)\Gamma^{T}X^{T}(t)\Lambda^{T})]\Lambda_{\beta }=[I_{N}⊗({\tilde{H}}^{T}(t)\Gamma^{T}X^{T}(t)\Lambda^{T})]\Lambda_{\beta }$$ (14)

Via the Eqs (12)–(14), one has

$$\begin{array}{l} {\dot{V}}_{1}(t)\leq (\beta +\gamma )e^{T}(t)e(t)+\frac{1}{2}\tilde{A}(t{)}^{T}(P+P^{T})\tilde{A}(t)+ce^{T}(t)[\Lambda ⊗(\Gamma \tilde{H}(t))]\tilde{A}(t)\\ \;-c{\tilde{A}}^{T}(t)[I_{N}⊗({\tilde{H}}^{T}(t)\Gamma^{T}X^{T}(t)\Lambda^{T})]\Lambda_{\beta }\}\\ \;=(\beta +\gamma )e^{T}(t)e(t)+\frac{1}{2}\tilde{A}(t{)}^{T}(P+P^{T})\tilde{A}(t)+ce^{T}(t)[\Lambda ⊗(\Gamma \tilde{H}(t))]\tilde{A}(t)\\ \;-c{\tilde{A}}^{T}(t)[\Lambda^{T}⊗({\tilde{H}}^{T}(t)\Gamma^{T})]e(t)\\ \;\leq (\beta +\gamma )e^{T}(t)e(t)+\frac{1}{2}\tilde{A}(t{)}^{T}(P+P^{T})\tilde{A}(t)<0 \end{array}$$ (15)

Therefore, the cluster synchronization error *e*(*t*) and edges’ state $\tilde{A}(t)$ are asymptotic stability via Lyapunov stability theory. This completes the proof of Theorem 1.

**Remark 3:** (i) The dynamics of edges (7) can be divided into two parts: one is the linear term, the other is the coupling term about the nodes’ state. The matrix form of edges system is proposed in [28–31] to discuss the tracking and synchronization control problem. In [31], a more simplified and intuitive linear equation is proposed, but a strong correlation is set to the reference targets between edges and nodes, meanwhile, the cluster synchronization is not considered. (ii) The result about the state of edges is asymptotic stability implies that the nodes connect with weak strength, when the cluster synchronization is achieved. The outer-coupling strength matrix (topology) are set to the every row-sum equal zero in the most existing results about cluster synchronization of CDNs, these results are not conflict with the conclusion in Theorem 1.

In the dynamics of edges (7), the network information, such as, matrix Γ, function $\tilde{H}(t)$ and coupling strength *c* are needed to know. In order to overcome this shortage, we propose the adaptive dynamics of edges to explore the auxiliary role for nodes by using the state of edges and nodes.

When the Assumption 1 is satisfied, there exists a positive constant *ρ* satisfying that $\|c[\Lambda ⊗\Gamma \tilde{H}(t)]\|\leq \rho$. The adaptive dynamics of edges is proposed as follows

$$\dot{\tilde{A}}(t)=\{\begin{array}{c} -\hat{\rho }(t)\frac{\tilde{A}(t)}{\left\|\tilde{A}(t)\right\|}\|\Lambda_{\alpha }x(t)\|,\|\tilde{A}(t)\|\neq 0\\ 0,\|\tilde{A}(t)\|=0 \end{array}$$ (16)

Where adaptive law $\dot{\hat{\rho }}(t)=\|\tilde{A}(t)\|\|\Lambda_{\alpha }x(t)\|$.

**Theorem 2:** If the Assumptions 1 and 2 are held, the cluster synchronization of nodes systems (3) can be realized via the nodes controller (6) and the coupling auxiliary role of edges system (16).

**Proof:** Consider the following Lyapunov candidate function

$$V_{2}(t)=\frac{1}{2}[e^{T}(t)e(t)+\tilde{A}{\left(t\right)}^{T}\tilde{A}(t)+{\left(\hat{\rho }(t)-\rho \right)}^{2}].$$ (17)

The derivative of *V*_2_(*t*) can be derived as follows via the controller (6) and the dynamics of edges (16)

$$\begin{array}{l} {\dot{V}}_{2}(t)=e^{T}(t)\dot{e}(t)+\tilde{A}(t{)}^{T}\dot{\tilde{A}}(t)+(\hat{\rho }(t)-\rho )\dot{\hat{\rho }}(t)\\ \;\leq (\beta +\gamma )e^{T}(t)e(t)+ce^{T}(t)[\Lambda ⊗(\Gamma \tilde{H}(t))]\tilde{A}(t)\\ \;+\tilde{A}(t{)}^{T}\dot{\tilde{A}}(t)+(\hat{\rho }(t)-\rho )\dot{\hat{\rho }}(t)\\ \;\leq (\beta +\gamma )e^{T}(t)e(t)+\rho \|e^{T}(t)\|\tilde{A}(t)-\hat{\rho }(t)\frac{\tilde{A}{\left(t\right)}^{T}\tilde{A}(t)}{\left\|\tilde{A}(t)\right\|}\|e(t)\|\\ \;+(\hat{\rho }(t)-\rho )\|\tilde{A}(t)\|\|e(t)\|\\ \;=(\beta +\gamma )e^{T}(t)e(t)+(\rho -\hat{\rho }(t))\|e^{T}(t)\|\|\tilde{A}(t)\|\\ \;+(\hat{\rho }(t)-\rho )\|\tilde{A}(t)\|\|e(t)\|\\ \;=(\beta +\gamma )e^{T}(t)e(t)\\ \;\leq 0. \end{array}$$ (18)

By the Lyapunov stability theory, the error *e*(*t*), the state of links $\tilde{A}(t)$ and error $\hat{\rho }(t)-\rho$ are stable. Because $\dot{e}(t)$ is bounded, the result about $\underset{t\to \infty }{\lim}e(t)=0$ is derived by the Barbalat lemma. This completes the proof of Theorem 2.

**Remark 4.** (i) The method of adaptive edge strategy is employed widely to help the realization of cluster synchronization of nodes. However, the strength values of edge matrix are still assumed to satisfy the dissipative condition (every row-sum equal zero), the coupling characteristics between nodes and edges is not reflected in the existing methods, the proposed dynamics of edges system (16) overcomes the defects. (ii) The results of Theorem 2 show that the state of edges is stable via the dynamics (16), this means that the interconnect term in nodes’ dynamic Eq (1) will not converge to zero when the cluster synchronization is achieved. This result is different from the most existing literatures, but the conclusion is more realistic, such as, the information is still exchanging after the synchronization of populations is realized.

### Simulation example

In this section, we use MATLAB for simulation experiments. Consider a 3-D neuron as the isolated node of network, whose dynamics is as follows

$${\dot{x}}_{i}(t)=-x_{i}(t)+(Y+Y_{i})G(x_{i}(t)),$$ (19)

where $x_{i}(t)={\left[x_{i1}(t),x_{i2}(t),x_{i3}(t)\right]}^{T}\in R^{3}$ is the state vector, $G(x_{i}(t))=[g(x_{i1}(t)),$ ${g(x_{i2}(t)),g(x_{i3}(t))]}^{T}$, g(s(t))=0.5(|s(t)+1|−|s(t)−1|), $Y_{i}=0.01*randn(1,1)*ones(3,3)$, and $Y=[\begin{array}{ccc} 1.25 & -3.2 & -3.2\\ -3.2 & 1.1 & -4.4\\ -3.2 & 4.4 & 1 \end{array}]$.

Remark 5. “*randn*” and “*ones*” are functions in MATLAB, “*randn(n*,*m)*” generates a *n×m* matrix of normal random distribution, “*ones(n*,*m)*” generates a *n×m* matrix with all elements are 1.

Let functions $f_{i}(x_{i}(t))=-x_{i}(t)+(Y+Y_{i})G(x_{i}(t))$, $h_{i}(x_{i}(t))=[cos(x_{i1}(t)x_{i2}(t)),$ $sin(x_{i2}(t)x_{i3}(t)),tan(x_{i3}(t)x_{i1}(t)){]}^{T}$ (obviously, the Assumption 2 is satisfied), inner coupled matrix Γ = *randn*(3), *c* = 1. In this simulation, we make ‖θ(x(t))‖=‖F(x(t))‖. The number of nodes is 20, i.e., *N* = 20, let *M* = 4, *G*_1_ = {1,2,3,4,5}, *G*_2_ = {6,7,8,9,10}, *G*_3_ = {11,12,13,14,15}, *G*_4_ = {16,17,18,19,20}, control gain *β* = −11, *γ* = 1, initial values x(0)=randn(3N,1), $\tilde{A}(0)=5*randn(N^{2},1)$. The matrix $P_{i}=P_{i3}P_{i1}P_{i3}{}^{T}$, in which $P_{i1}=diag\{p_{i1},p_{i2},p_{i3}\}$, $P_{i2}=randn(3)$, *p*_*i*1_,*p*_*i*2_,*p*_*i*3_ is arbitrary negative real number, *P*_*i*3_ is the orthogonal matrix of *P*_*i*2_, $\hat{\rho }(0)=randn(1)$.

**Fig 3:**
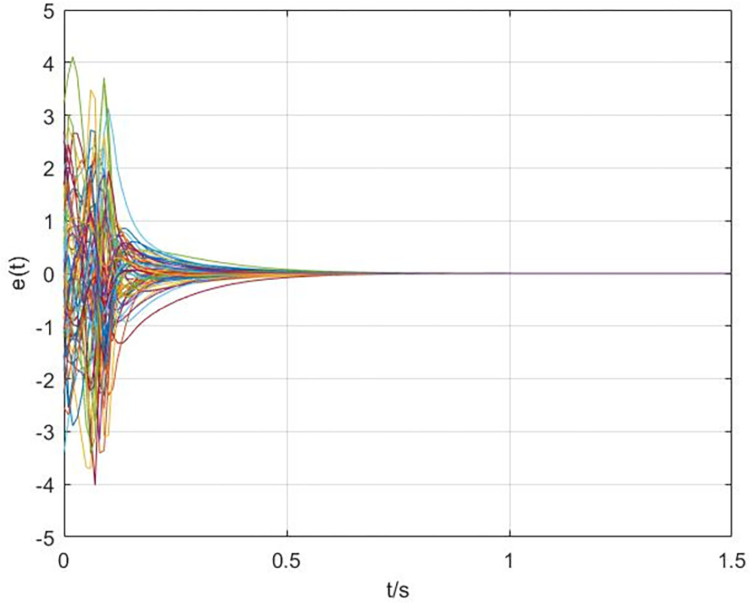
The response curves of error *e*(*t*) via the dynamics of edges (7).

**Fig 4:**
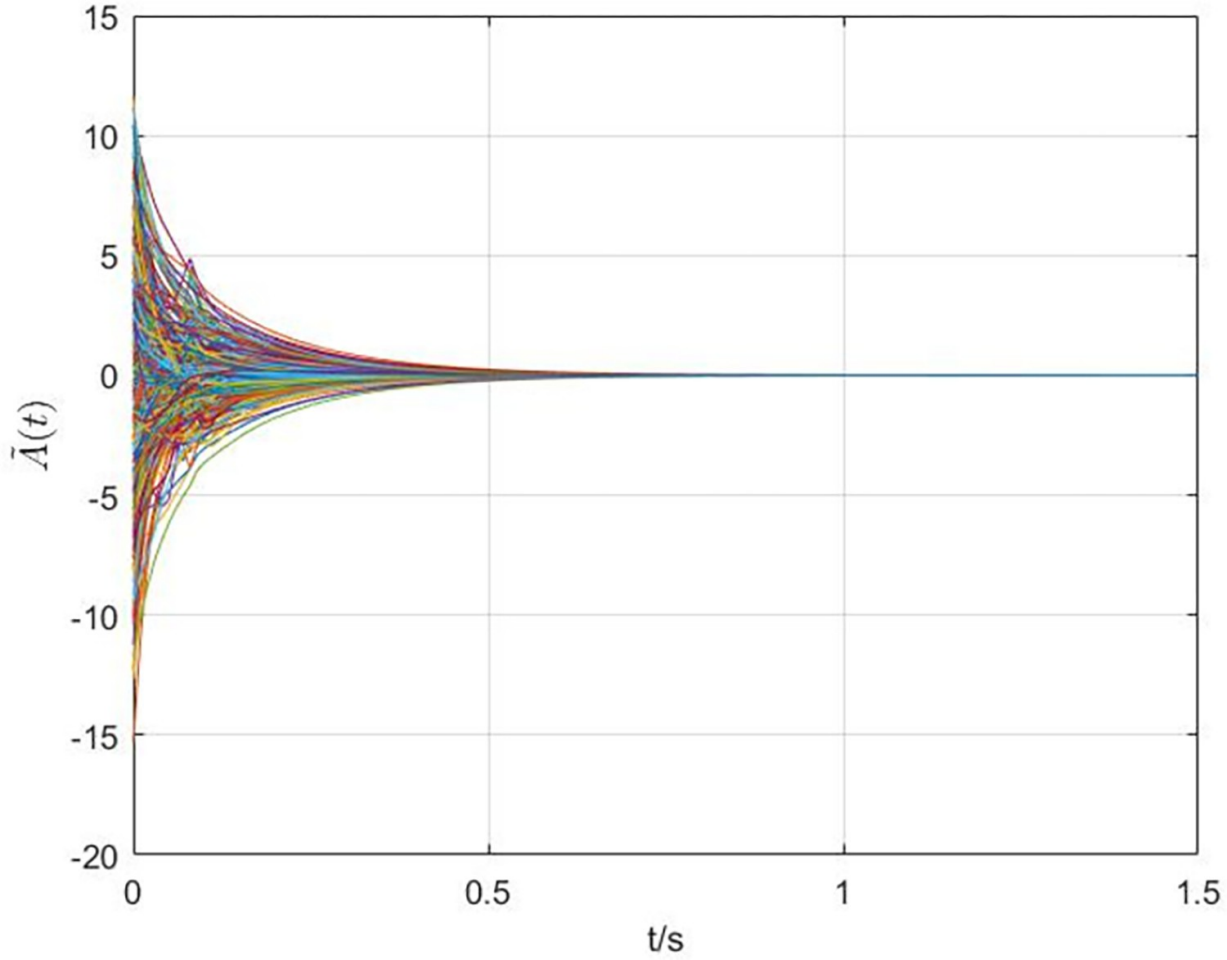
The response curves of state $\tilde{A}(t)$ of the dynamics of edges (7).

**Fig 5:**
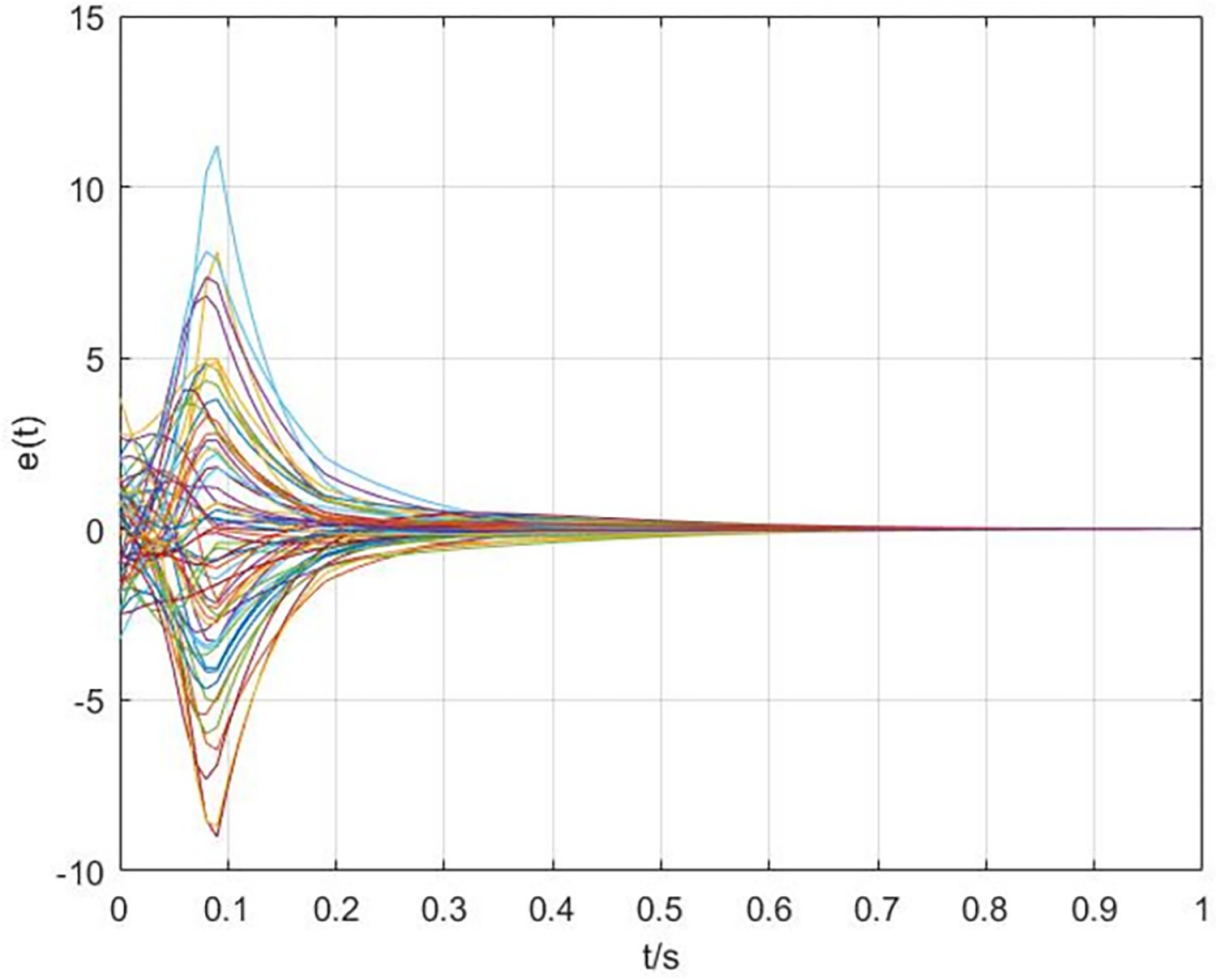
The response curves of error *e*(*t*) via the dynamics of edges (15).

**Fig 6:**
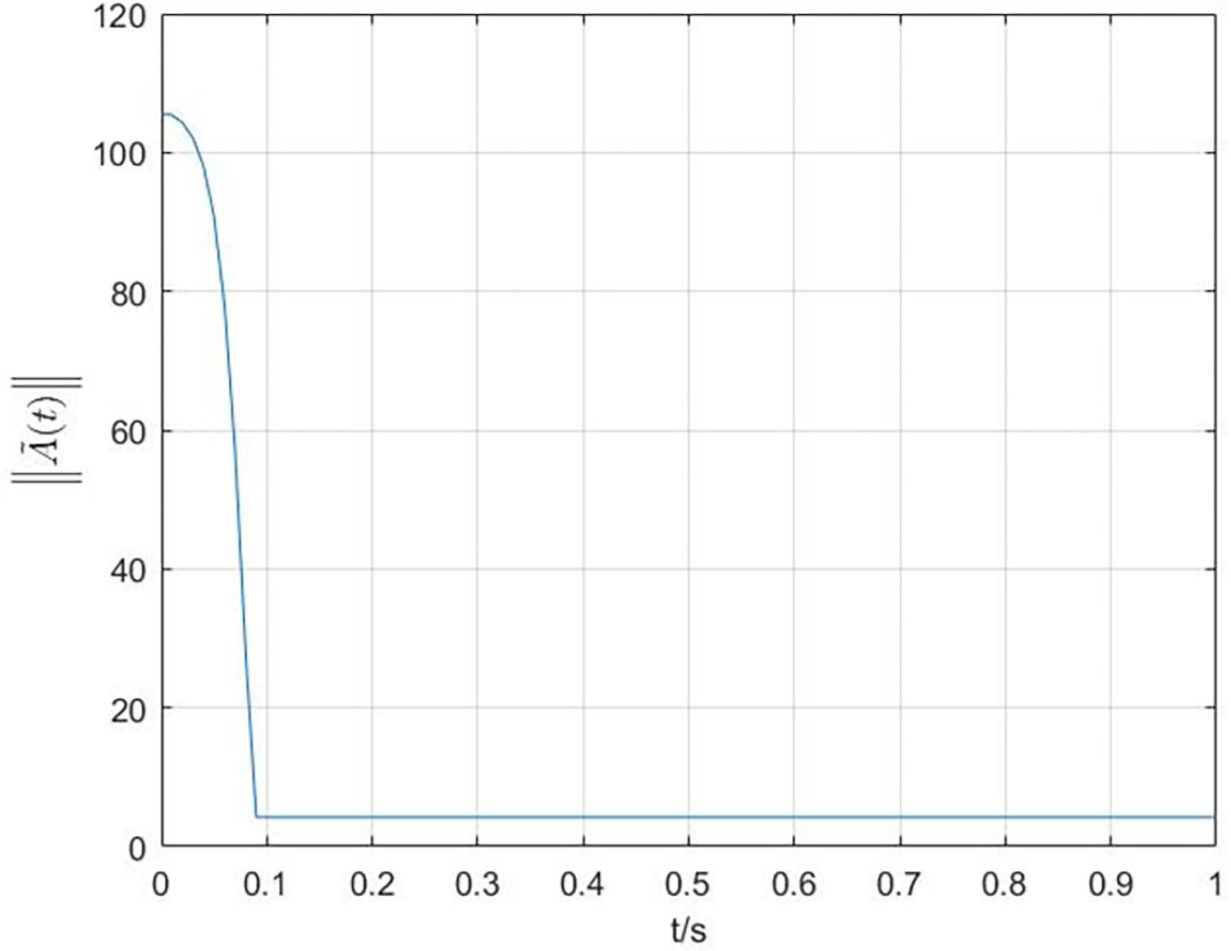
The response curves of state $\tilde{A}(t)$ of the dynamics of edges (15).

**Fig 7:**
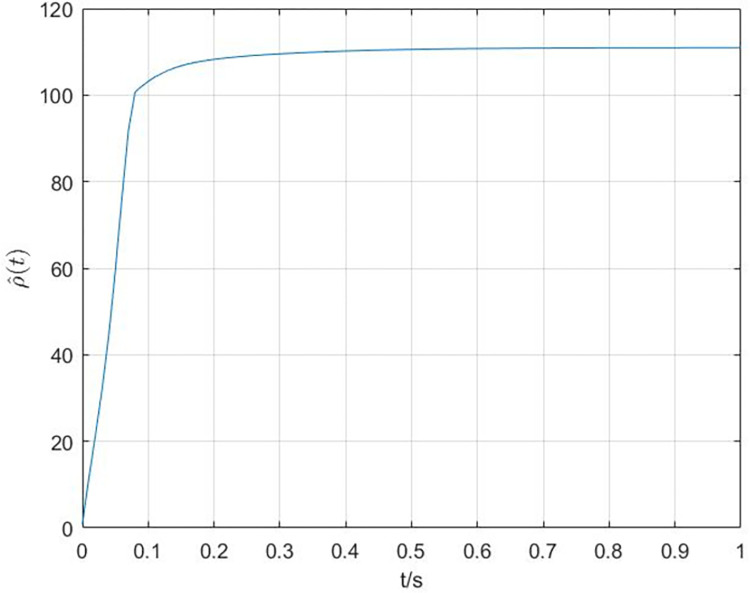
The response curves of $\hat{\rho }(t)$.

**Fig 8:**
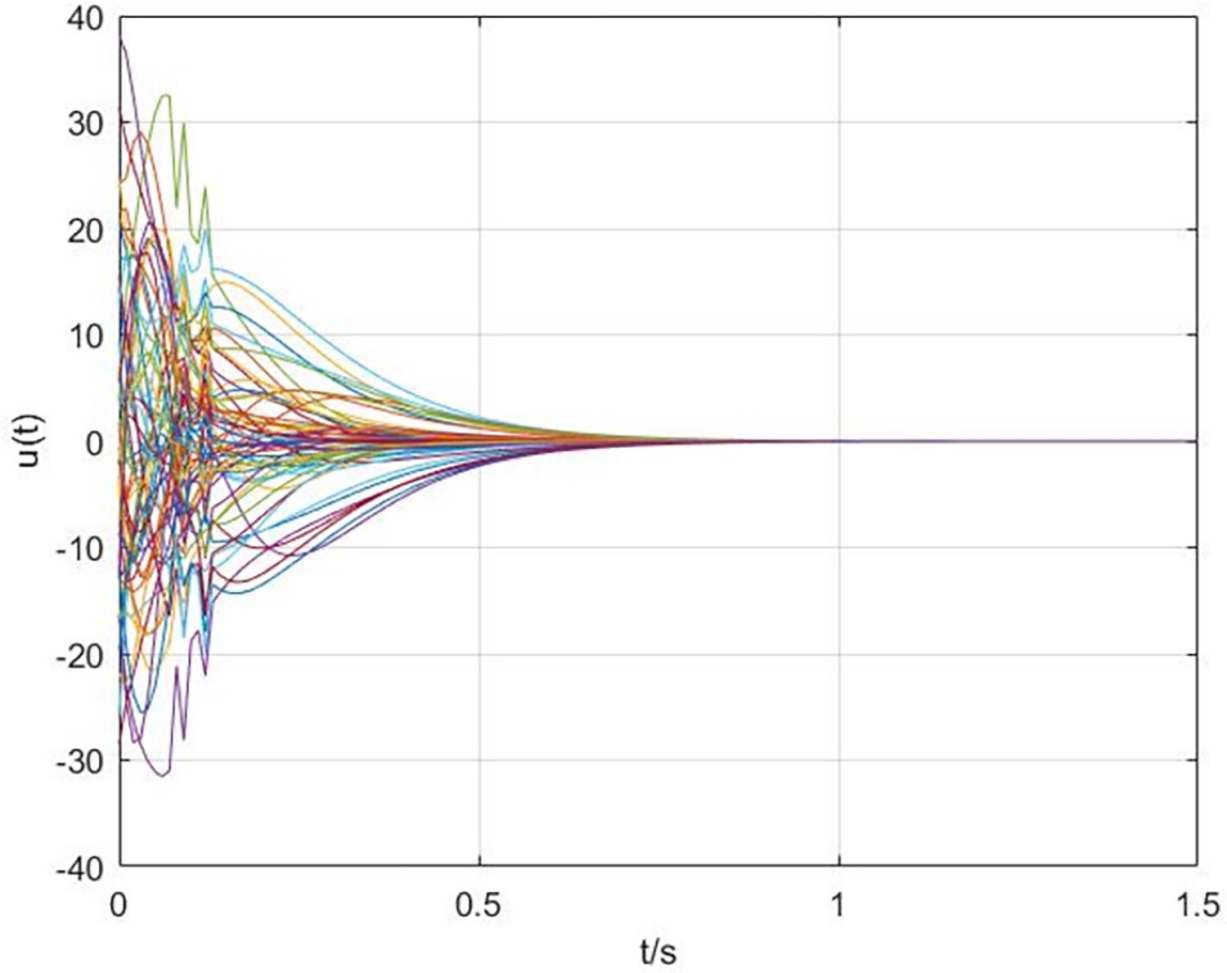
The response curves of controller *u*(*t*).

The simulation results are shown in Figs 3–8. In Figs 3 and 4, we can see that the cluster synchronization error is convergent, meanwhile, the state of edges is also asymptotically stable. These results illustrate the effectiveness of cluster synchronization control law (6) and dynamics (7). By using the dynamics of edges (15), the cluster synchronization of NS can be realized, the boundedness of $\tilde{A}(t)$ and $\hat{\rho }(t)$ is guaranteed from Figs 5–7. It should be noted that the state of links $\tilde{A}(t)$ is not asymptotically stable, this means the information exchanges between nodes still exist after the cluster synchronization is realized. Fig 8 shows the state of controller *u*(*t*) is stable, this simulation result is consistent with the conclusion of theoretical analysis.

## Conclusion

In this paper, two dynamics of edges are proposed to explore the cluster synchronization evolution mechanism of continuous-time CDNs. By the auxiliary role of linear edge dynamics, the cluster synchronization of the controlled nodes can be achieved, the state of edges is asymptotic stable. Via the adaptive dynamics of edges, the information exchanges between nodes still exist after the cluster synchronization of controlled nodes is realized, this conclusion is different from the most existing results, but it is more realistic. From the viewpoint large-scale system, this paper mainly explores the auxiliary mechanism of dynamic edges for the dynamical behaviors of nodes, meanwhile, provides a novel perspective for revealing the essential relation between structure and function in complex networks.

## Supporting information

S1 Data (RAR)
